# Supplementary material for: Patterns of Intron Gain and Loss in Fungi
Source: PLoS Biol. 2004 Nov 30;2(12):e422. doi: 10.1371/journal.pbio.0020422 (PMC532390; doi:10.1371/journal.pbio.0020422)
Supplement: Table S1 — Also available at http://genes.mit.edu/NielsenEtAl/. (4.3 MB ZIP). [file pbio.0020422.st001.zip › NielsenEtAl/html/596.html]

AN5452.1.NCU00396.1.MG06457.1.FG07245.1


```
 CLUSTAL W (1.82) Multiple Sequence Alignments - Introns Inserted


Sequence 1: NCU00396.1	1271 aa
Sequence 2: FG07245.1	1208 aa
Sequence 3: MG06457.1	1229 aa
Sequence 4: AN5452.1	1226 aa
Alignment Length: 1279 aa
Number Identitical Residues: 752 aa
Alignment Score (without introns) 35329


MG06457.1 	MATTSNMFLYSLSIQPPSTITRAILGQFSGTKEQQIVAASGSRLTLYRPDPTQGKVVPLM
NCU00396.1	MATTSNMFLYSLTIQPPTAVTQALLGQFSGTKEQQILTASGSRLTLLQPDPRQGKVNTLL
FG07245.1 	MATTSNMFLYSLTVQPPTNVTQAVLGQFAGTREQLIITGAGSQLSLLRPDPSQGKVITLL
AN5452.1  	MATTSHMFMYSLTIQPPTAITQAILGQFAGTKEQQIVTASGSKLTIHRPDPTQGKVIPLY
          	*****:**:***::***: :*:*:****:**:** *::.:**:*:: :*** **** .* 

MG06457.1 	SHDVFGIIRDLASFRLAGSSKD1YLIIASDSGRITIVEYLPAQNRFSRIHLETFGKSGVR
NCU00396.1	SHDIFGIVRAIASFRLAGSHKD1YIILATDSGRITIIEYLPKTNKFQRIHLETFGKSGVR
FG07245.1 	SHDVFGIIRSLAAFRLAGSNKD1YLIIASDSGRITIIEYLPAQNRFQRLHLETFGKSGVR
AN5452.1  	TQDVFGIIRTLAAFRLAGSNKD1YIIIGSDSGRITIIEYVPSQNRFNRIHLETFGKSGVR
          	::*:***:* :*:****** ** *:*:.:*******:**:*  *:*.*:***********

MG06457.1 	RVVPGQYLAADPKGRACLIASVERCKLVYVLNRNSQAELTISSPLEAHKNGTLTLSLVAL
NCU00396.1	RVIPGQYLAADPKGRACLISALEKNKLVYVLNRNSQAELTISSPLEAHKPGVLVLSLVAL
FG07245.1 	RVIPGEYLACDPKGRACLIASTEKNKLVYVLNRNSQAELTISSPLEAHKPGVLVISMVAL
AN5452.1  	RVVPGQYLAVDPKGRACLIASVEKNKLVYVLNRNSQAELTISSPLEAHKPQTLVYSVVAL
          	**:**:*** *********:: *: ************************  .*. *:***

MG06457.1 	DVGYSNPVFAALEVDYSEVDQDPKGDAAQNVETVLNYYELDLGLNHVVRKWFDVVDSTAS
NCU00396.1	DVGYANPVFAALELDYTDADQDPTGQAREEVETQLVYYELDLGLNHVVRKWSDTVDRTSS
FG07245.1 	DVGYSNPVFAALEIDYSEVDQDSTGQAMEELDTQLVYYELDLGLNHVVRKWSDPVDPTAS
AN5452.1  	DAGYENPVFAALEVDYSESDQDPTGRAYEEVEKLLVYYELDLGLNHVVRKWTDPVDRTSS
          	*.** ********:**:: ***..* * ::::. * *************** * ** *:*

MG06457.1 	MLFQVPGGSDGPSGVLVCGEENITYRHSNQDAFRVPIPRRKGATEDPSRKRNIVSGVMHK
NCU00396.1	LLFQVPGGNDGPSGVLVCGEENVTYRHSNQEAFRVPIPRRSGATEDPQRKRVIVSGVMHK
FG07245.1 	ILFQVPGGNDGPSGVLVCGEENITYRHSNQEAFRVAIPRRRGATEDPNRKRTIVSGIMHK
AN5452.1  	MLFQVPGGADGPSGVLVCAEDNITYRHSNQDAFRVPIPRRKGAMENPERKRCITAGVMHK
          	:******* *********.*:*:*******:****.**** ** *:*.*** *.:*:***

MG06457.1 	LKGSAGAFFFLLQTEDGDLFKVTIDMVEDAEGNPTGEVRRLKIKYFDTIPVSNNLCILKS
NCU00396.1	LKGSAGAFFFLLQTDDGDLFKVTIDMIEDSDGNPTGEVKRLKIKYFDTIPVATSLCILKS
FG07245.1 	LKGNTGAFFFLLQTDDGDLFKLSIDMIEDEEGNPTGEVKRLKVKYFDTVPVASSLCILKS
AN5452.1  	MRG---AFFFLLQTEDGDLFKLTLDMVEDDKGQLTGEVKGLKIKYFDTVPLASSLLILKS
          	::*   ********:******:::**:** .*: ****: **:*****:*::..* ****

MG06457.1 	GFLFVASEFGNHLFYQFEKLGDDDEELEFFSSDFPVDPKEPYEPVYFYPRPTENLALVES
NCU00396.1	GFLFAASEFGNHHFYQFEKLGDDDEELEFSSDDFPTDPTASYNPVYFHPRPLENLVLVES
FG07245.1 	GFLYVATQFGNYSFYQFEKLGDDDEELEFYSDDFPADPKASYEPVYFHPRPTENLALVES
AN5452.1  	GFLYVAAEGGNHHFYQFEKLGDDDEETEFNSDDFSADPAAPCTPVYFQPRGAENLNLVEA
          	***:.*:: **: ************* ** *.**..**  .  **** **  *** ***:

MG06457.1 	IDSMNPLMDLKVANLTEEDAPQIYTVSGKGARSTFRMLKHGLEVNEIVASQLPGTPSAVW
NCU00396.1	IDSMNPQVDCKVANLTGEDAPQIYSVCGNGARSTFRMLKHGLEVSEIVASELPGTPSAVW
FG07245.1 	IPAMNPLLDCKVANLTGEDAPQIYTICGNGPRSSFRMLKHGLEVNEIVASELPGIPSAVW
AN5452.1  	INSLNPLVDSKVVNISEDDAPQIFTVSGTGARSTFRTLKHGLEVSEIVESELPSVPSAVW
          	* ::** :* **.*:: :*****:::.*.*.**:** *******.*** *:**. *****

MG06457.1 	TTKLRRDDEYDAYIVLSFTNGTLVLSIGETVEEVSDTGFLSSVPTLAVQQLGDDGLVQVH
NCU00396.1	TTKLTKYDQYDAYIVLSFTNGTLVLSIGETVEEVSDSGFLTTAPTLAVQQMGEDGLIQVH
FG07245.1 	TLKLNRSEQYDAYIVLSFTNGTLVLSIGETVEEVSDSGFLTSVPTLAAQLLGDDGLIQVH
AN5452.1  	TTKLTRADEFDAYIVLSFANGTLVLSIGETVEEVTDTGFLSSAPTLAVQQLGEDSLIQIH
          	* ** : :::********:***************:*:***::.****.* :*:*.*:*:*

MG06457.1 	PKGIRHIRNGV-VNEWSSPQHRSIVAAATNERQVAVALSSGEIVYFEMDTDGSLAEYDEK
NCU00396.1	PKGIRHIVQGR-VNEWPAPQHRSIVAATANENQVVIALSSGEIVYFEMDSDGSLAEYDEK
FG07245.1 	PKGIRHIRNGN-VNEWAAPQHRSIVAATANAHQVAVALSSGEIVYFEMDADGSLAEYDEK
AN5452.1  	PRGIRHILADRRVNEWPAPQHRSIVAAATNERQVAVALSSGEIVYFELDADGSLAEYDER
          	*:*****  .  ****.:*********::* .**.:***********:*:*********:

MG06457.1 	KEMFGTVTSLSLGEVPEGRLRSSYLAVGCDDCTVRILSLDPESTLESKSVQALTAAPSAL
NCU00396.1	KEMSGTVTSLSVGQVPEGLKRSSFLAVGCDDCTVRILSLDPDSTLEMKSIQALTAAPSAL
FG07245.1 	KEMFGTVTCLSLGDVPEGRLRSSFLAVGCDDCTVRILSLDPESTLENKSVQALTAAPTSL
AN5452.1  	RQMSGTVTCLSLGEVPEGRVRSSFLAVGCDDSTVRILSLDPDTTLENKSVQALTAAPSAL
          	::* ****.**:*:****  ***:*******.*********::*** **:*******::*

MG06457.1 	SIMSMEDSSSGGTTLYLHIGLNSGVYLRTVLDEVTGELTDTRQKFLGPKAVRLFQVSVQK
NCU00396.1	SIMSMEDS-FGGSTLYLHIGLHSGVYLRTVLDEVTGELTDTRQKFLGPKPTRLFQVSVQD
FG07245.1 	AIIAMEDSSSGGSTLYLHIGLHSGVYLRTVLDEITGELTDTRQKFLGPKEVRLFQVTVQG
AN5452.1  	NIIAMADSSSGGTTLYLHIGLHSGVYLRTALDEVTGELSDTRTRFLGSKAVKLFQVSVTG
          	 *::* **: **:********:*******.***:****:*** :***.* .:****:*  

MG06457.1 	RTCVLALSSRSWLGFSDPVTKGFTMTPLNYEELEWGWNFVSEQCEEGMVGVNGQFLR2IF
NCU00396.1	QPCVLALSSRPWLGYTDPLTKGFMMTPLSYTELEYGWNFSSEQCLEGMVGIHANYLR2IF
FG07245.1 	KTCVLGLSSRPWLGYADPITKGFVVTPLNYVDLEWGWNFSSEQCEEGIVGIQGQSLR2IF
AN5452.1  	QTAVLALSSRPWLGYSDTQTKGFMLTPLDYVGLEWGWNFSSEQCVEGMVGIQGQNLR2IF
          	:..**.****.***::*. **** :***.*  **:**** **** **:**::.: ** **

MG06457.1 	AIEKLGDNVIQKSIPLTYTPRKLAKHPTQRIFYTIEADNNTLAPELREQLMAAPTAVNGD
NCU00396.1	SIEKLGDNMIQKSIPLTYTPKHLVKHPEQPYFYTIESDNNTLPPELRAKLLEQQ--SNGD
FG07245.1 	NIDRLGETLIQKSIPLTYTPKKLVKHPDQPLFYTIEADNNTLPPELRAQLLADPGVVNGD
AN5452.1  	SIEKLDNNMLQQSIPLAYTPRHFIKHPEEPLFYVIEADNNVLSPATRARLLEDSKARGGD
          	 *::*.:.::*:****:***::: *** :  **.**:***.*.*  * :*:      .**

MG06457.1 	ARVLPPDEFGYPRGNGRWASCISVVDPLGDGEELEPGVVQRIDLDNNEAALSMAVVSFAS
NCU00396.1	ATVLPPEDFGYPRAKGRWASCISIIDPISE----EPRVLQRIDLDNNEAAVSAAIVPFAS
FG07245.1 	SRVLPPEDFGYPKGTRRWASCINVIDPLSE----EGQVLQTIDLENNEAAVSAAIVPFSS
AN5452.1  	TTVLPPEDFGYPRGTGHWASCIQIIDPLDA-----KAVVGAVELEENEAAVSIAAVPFTS
          	: ****::****:.. :*****.::**:.        *:  ::*::****:* * *.*:*

MG06457.1 	QDGESFLVVGTGKDMVVNPRRFTEGYIHVYRFSEDGRELEFIHKTKVEEPPTALLPFQGR
NCU00396.1	QEGESFLVVGTGKDMVLDPRQFTEGYIHVYRFHEDGRDLEFIHKTRVEEPPLALIPFQGR
FG07245.1 	QDNESFLVIGTGKDMVVNPRSFSEGYLHIYRFLEGGRELEFIHKTKVEEPPLALLAFQGR
AN5452.1  	QDDETFLVVGTAKDMTVNPPSSAGGYIHIYRFQEDGKELEFIHKTKVEEPPLALLGFQGR
          	*:.*:***:**.***.::*   : **:*:*** *.*::*******:***** **: ****

MG06457.1 	LVAGIGRMLRIYDLGLRQLLRKAQAEVAPQLIVSLNTQGSRIIVGDVQHGLIYVAYKSET
NCU00396.1	LLAGVGKTLRIYDLGLKQLLRKAQADVTPTLIVSLQSQGNRIIVGDLQQGITYVVYKAEG
FG07245.1 	VLVAVGTSLRIYDLGMRQMLRKSQAEVATQQIVSLNTQGSRIIVGDVQQGVTYVVYKPAS
AN5452.1  	LLAGVGSVLRIYDLGMKQLLRKCQAAVAPKAIVGLQTQGSRIVVSDVRESVTYVVYKYQD
          	::..:*  *******::*:***.** *:.  **.*::**.**:*.*::..: **.**   

MG06457.1 	NRLIPFADDTIARWTTCTTMVDYDSTAGADKFGNLWILRCPEKASQESDEPGSEVHLVHS
NCU00396.1	NRLIPFADDTLNRWTTCTTMVDYESVAGGDKFGNIYIVRCPERVSQETDEPGSEIHLMHA
FG07245.1 	NKLIPFVDDTIARWTTCTTMVDYESVAGGDKFGNMFIVRCPEKASEEADEEQSGLHLINA
AN5452.1  	NVLIPFVDDSIARWTTAATMVDYETTAGGDKFGNLWLVRCPKKASEEADEEGSGAHLIHD
          	* ****.**:: ****.:*****::.**.*****::::***::.*:*:**  *  **:: 

MG06457.1 	RDYLHGTSNRLALMAHVYTQDIPTSICKTNLVVGGQEVLLWGGFQGTIGVLIPFVSREDA
NCU00396.1	RNYLHGTPNRLSLQVHFYTQDLPTSICKTSLVVGGQDVLLWSGLQGTVGVFIPFVSREDV
FG07245.1 	RDYLHGTPHRVSLMCHFYTQDIPTSITKASLVVGGQEVLLWSGIMGTIGVFIPFVSREDA
AN5452.1  	RGYLQGTPNRLELMIHVFTQDIPTSLHKTQLVAGGRDILVWTGFQGTIGILVPFVSREDV
          	*.**:**.:*: *  *.:***:***: *:.**.**:::*:* *: **:*:::*******.

MG06457.1 	DFFQSLEQHLRSEDPPLAGRDHLMYRGCYVPVKGVIDGDLCERYTMLPNDKKQMIAGELD
NCU00396.1	DFFQNLENHMRAEDPPLAGRDHLIYRGYYTPVKGVIDGDLCERFSLLPNDKKQMIAGELD
FG07245.1 	DFFQNLEQHLRTEDPPLAGRDHLMYRGYYAPVKGVIDGDLCERYNLLPNDKKQMIAGELD
AN5452.1  	DFFQSLEMQLASQCPPLAGRDHLIYRSYYAPVKGVIDGDLCEQYFLLSNDTKMMIAAELD
          	****.** :: :: *********:**. *.************:: :*.**.* ***.***

MG06457.1 	RSVREIERKIS0FHVNMA~YGDGVA1QLQLLSWKF---~-----------------~---
NCU00396.1	RSVREIERKIS0LGAPVV0LGNGEK~EGDIWDRGGLWE2NERLAIKVPFFCVASQI1TAD
FG07245.1 	RSVREIERKIS0Y-----~LGS---~------------~-----------------~---
AN5452.1  	RSVREIERKIS0VPAALA~LNVDLQ~DVSCLARSSALL~G----------------~---
          	***********         . .   . .          .                    

MG06457.1 	--------------------------
NCU00396.1	YSAATAQSPIVRERLLGLMDEAGHQV
FG07245.1 	--------------------------
AN5452.1  	--------------------------
          	
```
